# Platelets as Central Modulators of Post-Cardiac Arrest Syndrome: Mechanisms and Therapeutic Implications

**DOI:** 10.3390/biom16010134

**Published:** 2026-01-12

**Authors:** Chen-Hsu Wang, Jing-Shiun Jan, Chih-Hao Yang, Chih-Wei Hsia, Ting-Lin Yen

**Affiliations:** 1 Coronary Care Unit & General Cardiology, Cardiovascular Center, Cathay General Hospital, 280 Renai Rd., Sec. 4, Taipei 10630, Taiwan; chw@cgh.org.tw; 2 Department of Pharmacology, School of Medicine, College of Medicine, Taipei Medical University, No. 250, Wu Hsing St., Taipei 110301, Taiwan; d119101004@tmu.edu.tw (J.-S.J.); chyang@tmu.edu.tw (C.-H.Y.); 3 Department of Medical Research, Taipei Medical University Hospital, No. 252, Wu Hsing St., Taipei 110301, Taiwan; 4 Department of Medical Research, Cathay General Hospital, 280 Renai Rd., Sec.4, Taipei 10630, Taiwan

**Keywords:** cardiac arrest, post-cardiac arrest syndrome, platelets, thromboinflammation, platelet–leukocyte aggregates, therapeutics

## Abstract

Post-cardiac arrest syndrome (PCAS) remains a major cause of mortality and neurological impairment following successful resuscitation, yet the mechanisms linking global ischemia–reperfusion injury to microvascular and systemic dysfunction are not yet completely understood. While prior work has focused on inflammation, endothelial injury, and circulatory collapse, the central role of platelets in coordinating these pathological processes has not been comprehensively examined. This review provides the first integrated framework positioning platelets as core modulators, rather than secondary participants, in PCAS pathophysiology. We synthesize emerging evidence demonstrating that ischemia and reperfusion transform platelets into potent thromboinflammatory effectors through oxidative stress, DAMP-mediated pattern recognition signaling, and mitochondrial dysfunction. Hyperactivated platelets drive cerebral microthrombus formation, coronary no-reflow, and peripheral organ hypoperfusion, while platelet–leukocyte aggregates, neutrophil extracellular traps, and platelet-derived microparticles amplify systemic inflammation and endothelial injury. We further highlight the clinical significance of dynamic platelet dysfunction in coagulopathy, prognostication, and responses to post-arrest therapies including targeted temperature management and ECMO. Finally, we outline a novel, platelet-centered therapeutic paradigm, emphasizing selective interventions, such as GPVI inhibition, P-selectin blockade, FXI/XIa inhibition, and NETosis modulation, that target pathological platelet activity while preserving essential hemostatic function. In this review, by reframing platelets as the central determinants of PCAS, we report new mechanistic insights and therapeutic opportunities that are complementary to the existing post-arrest strategies and have the potential to improve survival and neurological outcomes after cardiac arrest.

## 1. Introduction

Cardiac arrest remains a major global health challenge, with survival rates having improved only modestly despite advances in resuscitation science and post-resuscitation care [1]. Even among patients who achieve the return of spontaneous circulation (ROSC), mortality and long-term neurological disability remain unacceptably high [2]. This poor prognosis is largely attributable to post-cardiac arrest syndrome (PCAS): a complex pathophysiological entity characterized by global ischemia–reperfusion injury, systemic inflammation, microcirculatory dysfunction, and multi-organ failure. Although these components have been extensively studied, the cellular mechanisms that orchestrate PCAS are not fully understood, and new therapeutic targets are urgently needed.

Platelets have traditionally been viewed through the lens of hemostasis and thrombosis; however, a growing body of evidence indicates that they are central regulators of inflammation, microvascular integrity, and immune responses [3,4]. Following cardiac arrest, the abrupt cessation of circulation induces severe metabolic derangement, cellular hypoxia, and blood stasis: conditions that prime platelets for activation. The subsequent reperfusion phase triggers a rapid burst of reactive oxygen species, the release of damage-associated molecular patterns (DAMPs), endothelial injury, and leukocyte recruitment, all of which synergistically amplify platelet activation [5,6]. This potent activation contributes not only to intravascular thrombosis but also to the propagation of inflammatory and immunologic responses that compound organ injury.

Accumulating evidence indicates that platelet-driven thromboinflammation is a key driver of cerebral microvascular obstruction, coronary no-reflow, and peripheral organ dysfunction after ROSC. Platelet–leukocyte aggregates, platelet-derived microparticles, and platelet-mediated neutrophil extracellular trap (NET) formations exacerbate endothelial dysfunction and capillary plugging, thereby amplifying the severity of brain and cardiac injuries [7,8]. Moreover, dynamic alterations in platelet count, reactivity, and phenotype after cardiac arrest correlate with clinical severity and neurological outcomes, suggesting a previously underappreciated prognostic and mechanistic role.

Despite this emerging understanding, platelet biology has not been fully integrated into the conceptual framework of PCAS, and the therapeutic potential of targeting platelet-mediated pathways remains largely unexplored. As new pharmacologic agents that are capable of modulating platelet activation, adhesion, and inflammatory signaling continue to emerge, revisiting the role of platelets in cardiac arrest is both timely and necessary.

In this review, we summarize the current evidence positioning platelets as central modulators of PCAS, focusing on mechanistic pathways that link ischemia–reperfusion injury, microcirculatory dysfunction, and systemic inflammation. We further highlight therapeutic strategies aimed at attenuating platelet-driven injury and discuss how integrating platelet-targeted interventions into post-arrest care may improve survival and neurological outcomes.

## 2. Pathophysiology of Platelet Activation in Cardiac Arrest

Cardiac arrest produces an abrupt cessation of systemic blood flow, initiating a cascade of biochemical and cellular events that profoundly alter the platelet biology. The transition from global ischemia to reperfusion generates an environment characterized by hypoxia, oxidative stress, endothelial disruption, and intense inflammatory signaling. These conditions synergistically transform circulating platelets from resting sentinels into potent amplifiers of thromboinflammation. Understanding the mechanisms that drive this transformation is critical in defining the role of platelets in post-cardiac arrest syndrome (PCAS). In this review, “platelet hyperactivation” refers to an early, heightened activation state characterized by amplified receptor signaling, granule secretion, and aggregation in response to ischemia–reperfusion stress. “Platelet dysfunction” denotes a later or severe pathological state in PCAS, marked by qualitative or quantitative impairment of platelet responses, including receptor desensitization, metabolic exhaustion, or consumptive thrombocytopenia. “Procoagulant platelets” specifically describe a distinct platelet subpopulation that exposes phosphatidylserine and exhibits an enhanced thrombin-generating capacity, thereby contributing disproportionately to coagulation and microvascular thrombosis.

### 2.1. Global Ischemia-Induced Platelet Priming

The ischemic phase of cardiac arrest creates a physiological milieu that primes platelets for activation, even before reperfusion begins. The cessation of blood flow leads to stagnant microcirculation, local acidosis, ATP depletion, and intracellular calcium overload—hallmarks of metabolic stress that sensitize platelets to subsequent activation [9]. Shear stress abnormalities and contact with the activated endothelium further promote the conformational activation of integrins such as αIIbβ3 and the upregulation of adhesion molecules, including the surface translocation of P-selectin [10]. Although this ischemia-induced priming may not fully trigger aggregation, it lowers the activation threshold, enabling an exaggerated response once reperfusion occurs [11].

### 2.2. Reperfusion-Driven Hyperactivation and Thromboinflammatory Signaling

Reperfusion following ROSC represents a critical inflection point in platelet activation. The sudden reintroduction of oxygen to hypoxic tissue generates a massive burst of reactive oxygen species (ROS), which modifies the platelet redox state and sensitizes the key signaling pathways [12]. Concurrently, the release of DAMPs—including HMGB1, mitochondrial DNA, and ATP [13,14]—from the injured myocardium, brain, and peripheral tissues engages the pattern recognition receptors on platelets: notably, Toll-like receptors TLR2 and TLR4 [15]. These signals converge on downstream effectors such as Syk, PI3K, and PLCγ2 [15], promoting rapid granule secretion, surface P-selectin expression [16], and integrin activation [14].

Platelet activation is further amplified by the engagement of collagen receptor GPVI, the C-type lectin receptor CLEC-2, and ADP-mediated P2Y_12_ signaling [17,18,19]. Together, these pathways drive robust platelet aggregation, procoagulant activity, and crosstalk with immune cells [14,16]. The resulting thromboinflammatory phenotype is characterized not only by enhanced adhesion and aggregation but also by the release of proinflammatory mediators that propagate systemic injury.

### 2.3. Platelet Mitochondrial Dysfunction as an Amplifier of Activation

Mitochondrial integrity is essential for normal platelet function, and mitochondrial perturbations during ischemia–reperfusion contribute substantially to post-arrest dysfunction. ROS overload, impaired electron transport, and mitochondrial membrane depolarization [20] promote the generation of mitochondrial-derived DAMPs and increase cytosolic calcium, further lowering the threshold for activation [21]. The mitochondria-dependent externalization of phosphatidylserine (PS) enhances platelet procoagulant activity [22] and fosters thrombin generation [23], while mitochondrial ROS support the sustained activation of the GPVI–Syk and P2Y_12_ pathways [24]. Importantly, mitochondrial dysfunction also contributes to the formation of procoagulant platelets and microparticles, linking metabolic dysregulation to microvascular thrombosis and inflammatory injury.

**Figure 1:**
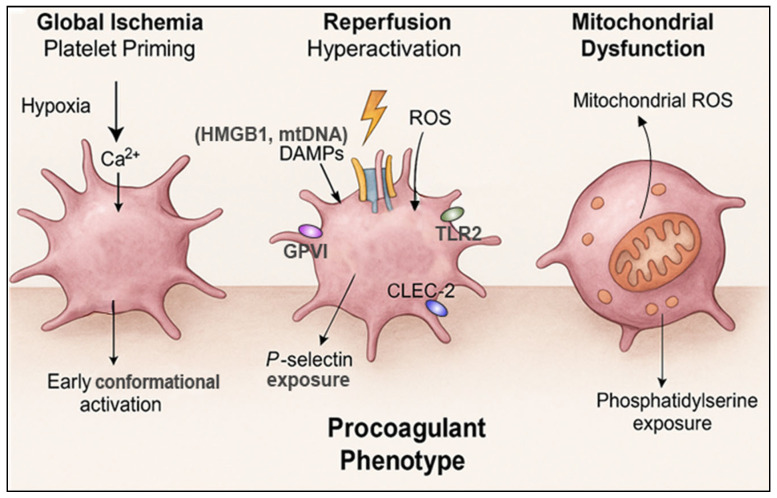
**Mechanisms of platelet activation during global ischemia and reperfusion.** Schematic representation of the sequential processes that transform platelets into thromboinflammatory effectors during cardiac arrest. Global ischemia induces hypoxia, intracellular calcium elevation, metabolic stress, and early conformational activation of platelets. Reperfusion triggers an oxidative burst and release of damage-associated molecular patterns (DAMPs, such as HMGB1 and mitochondrial DNA), activating Platelet glycoprotein VI (GPVI), C-type lectin-like receptor 2 (CLEC-2), and Toll-like receptors (TLR2) signaling pathways, leading to granule secretion and P-selectin exposure. Reperfusion-associated mitochondrial dysfunction increases mitochondrial ROS and causes loss of mitochondrial membrane potential, promoting phosphatidylserine exposure and the transition to a procoagulant platelet phenotype. These coordinated events establish the foundation for platelet-driven thromboinflammation in post-cardiac arrest syndrome.

Collectively, the ischemia–reperfusion environment transforms platelets into hyperreactive, prothromboinflammatory effectors. Through metabolic priming, receptor-mediated signaling, and mitochondrial dysfunction, platelets are emerging as key initiators and amplifiers of the pathological processes that define PCAS. These mechanistic insights lay a foundation for understanding how activated platelets drive microvascular injury, systemic inflammation, and adverse clinical outcomes downstream following cardiac arrest (Figure 1).

### 2.4. Temporal Evolution of Platelet Phenotypes After Cardiac Arrest

Platelet responses in PCAS are dynamic and evolve across distinct temporal phases following cardiac arrest. In the early reperfusion phase, spanning minutes to hours after ROSC, platelets undergo rapid hyperactivation driven by oxidative stress, DAMP-mediated pattern recognition signaling, endothelial disruption, and abrupt changes in shear stress. During this stage, platelets exhibit enhanced receptor signaling (e.g., GPVI, P2Y_12_), increased granule secretion, surface P-selectin expression, and augmented aggregation and procoagulant activity. This hyperactivated phenotype contributes to early microvascular thrombosis, platelet–leukocyte aggregate formation, and the initiation of thromboinflammatory cascades.

As PCAS progresses into later phases, sustained activation, metabolic stress, and ongoing endothelial injury promote a transition toward platelet dysfunction and exhaustion. This stage is characterized by receptor desensitization or shedding (e.g., GPVI, GPIbα), mitochondrial dysfunction, impaired aggregation responses, and consumptive thrombocytopenia. Qualitative platelet defects may coexist with paradoxical circuit- or inflammation-driven hypercoagulability, particularly in patients undergoing targeted temperature management or ECMO. Together, these temporal shifts underscore that platelet hyperactivation and platelet dysfunction are not mutually exclusive but represent sequential, context-dependent phenotypes that shape thrombotic risk, bleeding vulnerability, and therapeutic responsiveness throughout the post-arrest course.

## 3. Role of Platelets in Microcirculatory Dysfunction After ROSC

Microcirculatory dysfunction is a defining feature of post-cardiac arrest syndrome and plays a decisive role in determining neurological and cardiovascular outcomes [25,26]. Although global ischemia–reperfusion injury has long been recognized as the main initiator of microvascular damage, emerging evidence highlights platelets as central mediators of capillary obstruction, endothelial injury, and regional hypoperfusion. Hyperactivated platelets generated during and after reperfusion interact with leukocytes, fibrin, and dysfunctional endothelium to form microthrombi that impede tissue perfusion long after systemic circulation has been restored. This section outlines how platelet-driven processes contribute to cerebral, cardiac, and peripheral organ microcirculatory failure following cardiac arrest.

### 3.1. Cerebral Microthrombi and Hypoxic–Ischemic Brain Injury

The brain is uniquely vulnerable to disturbances in microvascular patency after cardiac arrest. Even when global hemodynamics appear to be restored, multiple studies have demonstrated persistent reductions in cerebral blood flow, which is often referred to as “delayed cerebral hypoperfusion” [27,28]. Platelets are pivotal contributors to this phenomenon. Reperfusion promotes the formation of platelet-rich microthrombi within the cerebral microvasculature, driven by ROS-enhanced GPVI signaling, P-selectin-mediated leukocyte recruitment, and the exposure of subendothelial collagen due to reperfusion-induced endothelial injury [29,30].

These microthrombi occlude capillaries and arterioles, aggravating the mismatch between metabolic demand and oxygen delivery. In addition, platelet–neutrophil aggregates and neutrophil extracellular traps (NETs) amplify capillary plugging, while platelet microparticles propagate local inflammation and endothelial activation [31]. The cumulative effect is an exacerbated hypoxic–ischemic brain injury, blood–brain barrier disruption, and increased vulnerability to secondary neurological damage. Clinically, the degree of platelet activation correlates with biomarkers of neuronal injury and allows for the prediction of adverse neurological outcomes [32].

### 3.2. Coronary Microvascular Obstruction and Post-Arrest Myocardial Dysfunction

Beyond the brain, platelets critically influence myocardial microcirculatory integrity after cardiac arrest. The phenomenon of “coronary no-reflow”, which is well-characterized in ischemia–reperfusion settings such as ST-elevation myocardial infarction, is becoming increasingly recognized in post-arrest patients [33,34]. Platelet hyperactivation promotes adhesion to the injured endothelium, aggregation within microvessels, and the formation of fibrin–platelet plugs that obstruct coronary microcirculation despite the restoration of epicardial flow [6].

Platelet-derived serotonin, thromboxane A_2_, and proinflammatory cytokines further contribute to microvascular spasms, endothelial dysfunction, and reperfusion arrhythmogenesis [35,36,37,38]. In addition, platelet–monocyte aggregates trigger tissue factor expression and downstream thrombin generation, enhancing microthrombus formation [39]. The resulting impairment in myocardial oxygen delivery worsens post-arrest myocardial stunning and contributes to global cardiac dysfunction: a major component of PCAS associated with early mortality.

### 3.3. Peripheral Organ Microcirculatory Failure and Multi-Organ Dysfunction

Microvascular obstruction extends beyond the brain and heart to involve peripheral organs, contributing to the systemic pathophysiology of PCAS. The platelet-mediated plugging of renal microvessels promotes acute kidney injury: a frequent and prognostically significant complication following ROSC [40]. In the liver, sinusoidal platelet accumulation disrupts microvascular flow and exacerbates ischemic hepatocellular injury [41]. Pulmonary microthrombi and platelet–neutrophil interactions contribute to acute lung injury through increased capillary permeability and inflammatory cell infiltration [42,43].

A critical upstream driver of this widespread dysfunction is endothelial glycocalyx degradation, which occurs rapidly during reperfusion [44]. A loss of glycocalyx integrity exposes adhesion molecules and basement membrane components, markedly increasing platelet adhesion and activation [45]. Importantly, platelet activation and endothelial glycocalyx degradation in PCAS are best viewed as a bidirectional process, rather than a linear cause–effect relationship. Early ischemia–reperfusion-induced glycocalyx loss facilitates platelet adhesion and activation, while activated platelets, through the release of inflammatory mediators, proteases, and reactive oxygen species, can further exacerbate glycocalyx shedding, establishing a self-amplifying feedback loop. This creates a self-perpetuating cycle of endothelial injury, platelet recruitment, and microvascular occlusion, amplifying multi-organ failure in PCAS [46].

**Figure 2:**
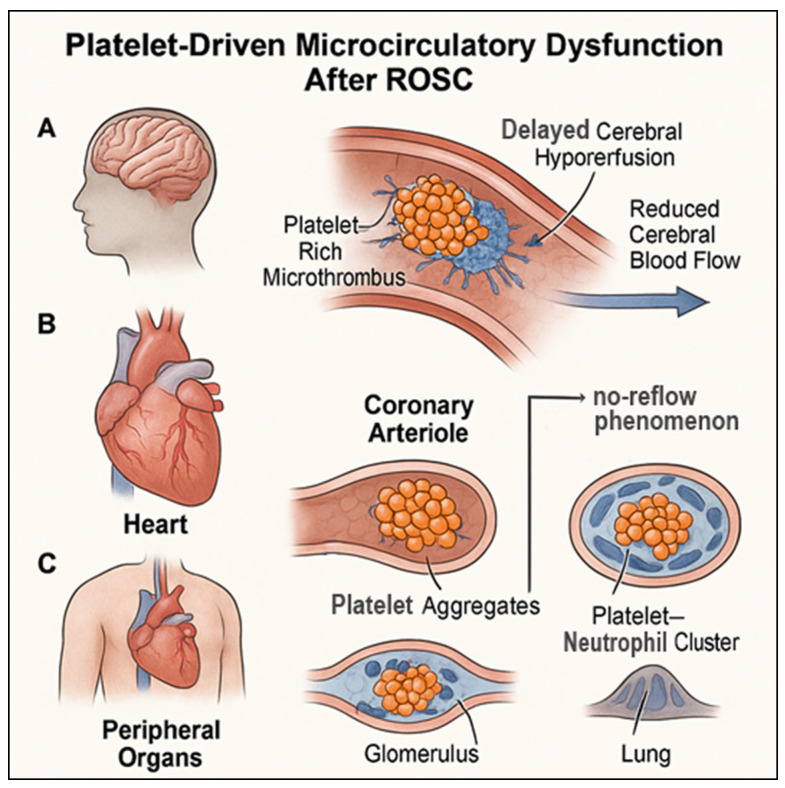
**Platelet-driven microcirculatory dysfunction after return of spontaneous circulation.** Illustration of how platelet hyperactivation contributes to microvascular obstruction across multiple organs following cardiac arrest. (**A**) In the brain, platelet-rich microthrombi occlude small vessels, leading to delayed cerebral hypoperfusion and reduced cerebral blood flow. (**B**) In the heart, platelet aggregates within coronary arterioles contribute to the coronary no-reflow phenomenon and exacerbate post-arrest myocardial dysfunction. (**C**) In peripheral organs, including the kidney, liver, and lung, platelet aggregates and platelet–neutrophil clusters impair the microcirculatory flow, promoting organ injury and multi-organ dysfunction. Together, these mechanisms highlight platelet-mediated microvascular obstruction as a central driver of post-cardiac arrest syndrome.

Across diverse PCAS presentations, platelets often act as central architects; platelets are central architects of microcirculatory dysfunction after cardiac arrest, orchestrating a complex interplay among thrombosis, inflammation, and endothelial injury. By driving cerebral hypoperfusion, coronary no-reflow, and peripheral organ damage, hyperactivated platelets link ischemia–reperfusion injury to the multi-organ manifestations of PCAS. These mechanistic insights underscore the need to integrate platelet biology into conceptual models of post-arrest care and highlight new avenues for targeted therapeutic interventions (Figure 2).

## 4. Platelet–Leukocyte Interactions and Systemic Inflammation

Systemic inflammation is a defining component of PCAS and is increasingly recognized as a major contributor to neurological injury, myocardial dysfunction, and multi-organ failure [47]. Beyond their well-established roles in thrombosis, platelets are potent immunomodulatory cells that are capable of amplifying inflammatory responses through direct interaction with leukocytes and the release of bioactive mediators. Following cardiac arrest, the combination of global ischemia, endothelial injury, and reperfusion-associated DAMP release creates an environment that rapidly mobilizes platelets as key participants in the innate immune response. This section describes the mechanisms by which platelet–leukocyte interactions shape the inflammatory landscape of PCAS. Importantly, platelet–leukocyte interactions in PCAS are unlikely to be uniform across patients. Arrest etiology (cardiac versus non-cardiac), duration of no-flow and low-flow intervals, and pre-existing comorbidities such as sepsis or atherosclerosis may substantially modulate the magnitude, timing, and clinical consequences of platelet-driven thromboinflammatory responses.

### 4.1. Platelet–Monocyte Aggregates: Drivers of Tissue Factor Expression and Inflammasome Activation

Platelet–monocyte aggregates are among the most sensitive markers of platelet activation and are markedly increased following ROSC [48,49]. The engagement of P-selectin on activated platelets with PSGL-1 on monocytes fosters stable aggregate formation, which triggers profound immunologic consequences. Activated platelets upregulate tissue factor (TF) expression on monocytes through CD40–CD40L [50] and thrombin–PAR signaling [51], thereby linking inflammation to the initiation of coagulation. This TF induction promotes thrombin generation, exacerbating microvascular thrombosis and further amplifying platelet activation in a feed-forward loop.

Moreover, monocytes engaged by platelets exhibit increased activation of the NLRP3 inflammasome, leading to the secretion of IL-1β and IL-18 [52]. These cytokines propagate systemic inflammation, endothelial activation, and vascular permeability—all hallmarks of PCAS. The degree of platelet–monocyte interaction correlates with organ dysfunction and mortality in inflammatory and ischemia–reperfusion settings, suggesting a similar significance in cardiac arrest.

### 4.2. Platelet–Neutrophil Aggregates and the Promotion of NETosis

Platelet–neutrophil interactions play a decisive role in shaping post-arrest inflammation and microvascular obstruction [53]. Activated platelets bind to neutrophils via P-selectin/PSGL-1 and integrin-dependent mechanisms, initiating the release of neutrophil extracellular traps (NETs) [54]. NETs consist of DNA fibers decorated with histones and proteases and act as prothrombotic scaffolds that trap platelets, erythrocytes, and fibrin, promoting extensive microvascular occlusion. Mechanistic insight is largely derived from stroke/sepsis/IR models.

In the context of cardiac arrest, NETs have been implicated in impaired cerebral perfusion, endothelial cell injury [53], and heightened coagulopathy. Platelet-induced NETosis is driven by HMGB1 [55,56], thrombin, and mitochondrial-derived ROS, all of which are abundant during reperfusion. NET components, including histone H3 [57] and extracellular DNA [58], further activate platelets and the coagulation cascade, creating a vicious cycle of inflammation-driven thrombosis. This process also contributes to the “no-reflow” phenomenon in the brain and heart.

### 4.3. Platelet-Derived Microparticles: Disseminated Amplifiers of Inflammation and Coagulation

Platelet-derived microparticles (PMPs) are small vesicles released from activated or apoptotic platelets [59], and their levels surge following cardiac arrest [60]. PMPs carry phosphatidylserine (PS), TF [61], mitochondrial fragments [62], and a rich repertoire of cytokines, chemokines [63], and microRNAs [62]. These vesicles disseminate throughout the circulation, exerting procoagulant, proinflammatory, and endothelial-disruptive effects.

PMPs potentiate thrombin generation [64], propagate inflammation by activating TLR pathways [65], and promote endothelial apoptosis [66]. Elevated PMP levels are associated with poor neurological outcomes in ischemic stroke, trauma, and critical illness [67,68,69], suggesting similar relevance in PCAS. By functioning as mobile platforms for the transfer of damage signals, PMPs extend the spatial footprint of platelet-mediated injury far beyond the initial site of activation.

**Figure 3:**
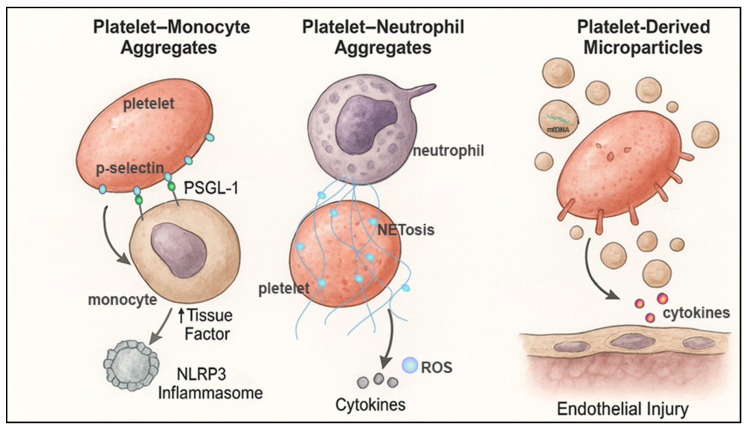
**Platelet–leukocyte interactions driving systemic inflammation in post-cardiac arrest syndrome.** Activated platelets engage multiple leukocyte populations to amplify systemic inflammation following cardiac arrest. Platelet–monocyte aggregates form through P-selectin–P-selectin glycoprotein ligand-1 (PSGL-1) binding, inducing monocyte tissue factor expression and activating the NOD-like receptor protein 3 (NLRP3) inflammasome. Platelet–neutrophil aggregates promote NETosis, generating extracellular DNA webs, ROS, and proinflammatory cytokines that contribute to microvascular obstruction. Platelet-derived microparticles release mitochondrial DNA and inflammatory cytokines and directly injure the endothelium. Collectively, these platelet–immune interactions constitute key mechanisms of immunothrombosis that exacerbate post-cardiac arrest syndrome.

Platelet–leukocyte interactions represent a crucial intersection between coagulation and innate immunity in PCAS. Through the formation of platelet–monocyte and platelet–neutrophil aggregates and the release of potent microparticles, activated platelets orchestrate a multifaceted inflammatory response that drives microvascular obstruction, endothelial injury, and organ dysfunction. These immunothrombotic mechanisms highlight platelets as central amplifiers of the systemic inflammatory milieu that characterizes PCAS and underscores the therapeutic potential of targeting platelet–immune cell interactions in post-arrest care (Figure 3).

## 5. Clinical Implications of Platelet Dysfunction in Post-Cardiac Arrest Syndrome

Platelet activation and dysfunction after cardiac arrest are not merely epiphenomena of global ischemia–reperfusion injury; rather, they have direct prognostic and therapeutic relevance. Dynamic alterations in platelet count, phenotype, and reactivity reflect the severity of systemic injury and contribute to the complex coagulopathy observed in PCAS. These changes correlate with microvascular dysfunction, inflammatory burden, and clinical outcomes, providing a mechanistic and measurable link between platelet biology and patient prognosis. Understanding these clinical implications is essential for identifying high-risk patients and refining post-arrest management strategies.

### 5.1. Coagulopathy and Hemostatic Dysregulation

Coagulation abnormalities are common following ROSC and reflect the intricate interplay among hyperactive platelets, consumptive coagulopathy, and endothelial injury. Early after ROSC, many patients exhibit transient thrombocytopenia resulting from platelet adhesion, sequestration, and consumption within microthrombi [70]. Concurrently, platelet function may be paradoxically impaired despite evidence of systemic activation, reflecting receptor desensitization, receptor shedding, or metabolic exhaustion after massive activation.

Thromboelastography (TEG) and rotational thromboelastometry (ROTEM) frequently reveal patterns that are consistent with hypercoagulability, including shortened clotting times and increased clot strength, driven in part by platelet procoagulant activity [71]. However, subsets of patients develop a hypocoagulable phenotype associated with platelet exhaustion, fibrinolytic shutdown, or systemic inflammation [72]. These hemostatic disturbances are clinically significant: both hypercoagulability and hypocoagulability are linked to increased mortality, refractory shock, and poor neurological recovery [73].

### 5.2. Platelet Biomarkers as Prognostic Indicators

Changes in platelet activation markers and morphometric indices offer valuable prognostic insights in PCAS. The mean platelet volume (MPV) and platelet distribution width (PDW)—reflecting the production of larger, more reactive platelets—are elevated in the post-arrest period and correlate with neurological injury and mortality [74]. Soluble P-selectin and CD40 ligand (sCD40L) levels [75] rise rapidly after ROSC, mirroring both endothelial and platelet activation. Elevated levels of these biomarkers are associated with greater systemic inflammation, more severe cerebral injury, and a higher likelihood of unfavorable neurological outcomes.

Platelet–leukocyte aggregates (PLAs), among the most sensitive markers of in vivo platelet activation, are markedly increased in PCAS and correlate with the lactate levels, organ dysfunction scores, and mortality [48,76,77]. Similarly, platelet-derived microparticle (PMP) concentrations correlate with inflammatory severity and cerebral injury biomarkers. These markers may help stratify PCAS severity and identify patients who are at high risk for microvascular complications or refractory inflammation, although their prognostic performance is likely influenced by arrest characteristics and underlying comorbidities.

These markers are primarily derived from observational human PCAS studies and should be interpreted as prognostic associations rather than causal mediators.

### 5.3. Interactions with Post-Resuscitation Therapies

Platelet dysfunction also affects responses to commonly used post-arrest therapies, including targeted temperature management (TTM) and extracorporeal membrane oxygenation (ECMO). Both therapies exert profound, often bidirectional influences on platelet activation, aggregation, receptor signaling, and survival, thereby shaping the trajectory of PCAS.

Hypothermia, even within the clinically applied range of 32–36 °C, disrupts platelet adhesion, aggregation, and receptor signaling [78,79,80]. Cooling alters platelets’ membrane fluidity [81]. TTM also delays the absorption and metabolic activation of oral P2Y_12_ inhibitors, due to reduced gastrointestinal motility [82] and hepatic perfusion [83,84], leading to weakened and delayed antiplatelet effects during the critical early hours of post-resuscitation care. Thus, TTM creates a paradoxical milieu: while hypothermia is neuroprotective [85], it simultaneously generates an acquired platelet dysfunction state that complicates the interpretation of coagulation assays, creates challenges regarding the dosing of antithrombotics, and may influence neurological and cardiac outcomes.

ECMO introduces an additional layer of platelet dysregulation. Because VA ECMO is the predominant modality used in refractory cardiac arrest and post-resuscitation cardiogenic shock, the platelet-related mechanisms discussed below are mainly relevant to VA rather than VV ECMO. Contact between blood and artificial circuit surfaces triggers continuous platelet activation, consumption, and eventual exhaustion [86]. Shear stress within the pump and oxygenator promotes von Willebrand factor unfolding and the loss of high-molecular-weight multimers that are essential for platelet tethering, while sustained activation increases P-selectin expression and platelet–leukocyte aggregate formation [87,88]. The result is a distinctive combination of thrombocytopenia and qualitative platelet defects coexisting with circuit-level hypercoagulability. Importantly, these effects are more pronounced in VA ECMO, which exposes platelets to higher shear stress and non-physiological arterial flow patterns, whereas VV ECMO predominantly affects venous circulation and is associated with a different thrombotic bleeding profile. Clinically, this dysregulated platelet state complicates anticoagulation management. Heparin requirements often fluctuate due to the consumption of antithrombin, while microthrombi may form within the circuit despite therapeutic anticoagulation. Conversely, ECMO-induced thrombocytopenia and qualitative defects heighten bleeding risks, especially during cannulation, limb ischemia interventions, or therapeutic hypothermia. The interface between ECMO-driven platelet alterations and PCAS-associated endothelial injury likely amplifies microcirculatory dysfunction, contributing to organ failure and refractory shock.

Accordingly, extrapolation from VV ECMO studies to PCAS should be made with caution, given the distinct hemodynamic and platelet-related features of VA ECMO.

These interactions highlight the need for individualized, physiology-guided antithrombotic management. Modulating platelet activity may reduce microvascular obstruction and inflammation, yet excessive antiplatelet therapy can cause hemorrhage in patients undergoing TTM, ECMO cannulation, or invasive procedures. A deeper understanding of therapy–platelet dynamics is essential in optimizing outcomes and underscores platelets as the central modulators of post-resuscitation pathophysiology.

**Figure 4:**
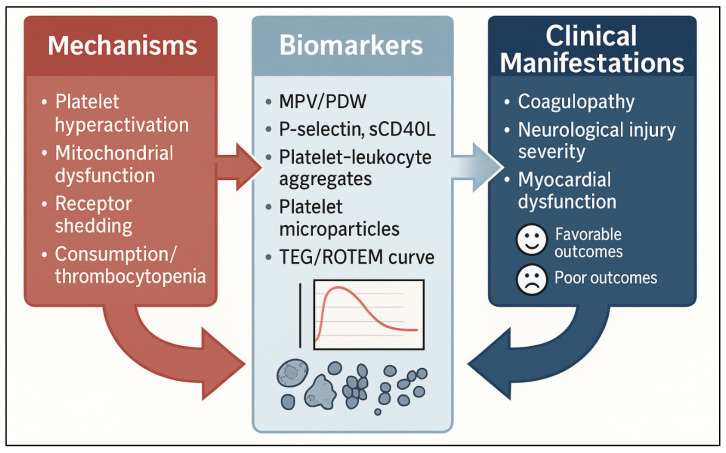
**Platelet dysfunction, biomarkers, and clinical impact in PCAS.** Mechanistic platelet abnormalities after cardiac arrest, hyperactivation, mitochondrial dysfunction, receptor shedding, and consumption lead to measurable biomarkers (mean platelet volume (MPV)/platelet distribution width (PDW), P-selectin, sCD40L, platelet–leukocyte aggregates, microparticles, and thromboelastography (TEG)/rotational thromboelastometry (ROTEM) changes) and manifest clinically as coagulopathy, neurological injury, and myocardial dysfunction. These platelet-driven disturbances influence the overall outcomes in post-cardiac arrest syndrome.

Platelet dysfunction in PCAS has major clinical implications, influencing coagulopathy, prognostic assessment, and responsiveness to essential post-resuscitation therapies. Platelet-based biomarkers offer promising avenues for early risk stratification, while understanding therapy–platelet interactions is critical in optimizing clinical management. These insights emphasize that platelets are not only mechanistic drivers of PCAS but also clinically relevant determinants of patient outcomes (Figure 4).

As summarized in Table 1, several platelet activation markers and cellular interactions discussed in this section are supported by direct observations from human PCAS cohorts, whereas others remain extrapolated from related ischemia–reperfusion conditions.

## 6. Therapeutic Strategies Targeting Platelets in Post-Cardiac Arrest Syndrome

The recognition of platelets as central contributors to microvascular obstruction, inflammation, and organ dysfunction in PCAS provides a rationale for adjunctive therapeutic interventions that modulate platelet activation and platelet–immune interactions. However, the therapeutic landscape remains challenging. Patients recovering from cardiac arrest frequently exhibit dynamic changes in platelet function, and any strategy aimed at attenuating platelet activity must balance the potential benefits of reducing thromboinflammation against the risks of bleeding, hemodynamic instability, or interference with post-resuscitation procedures. Nonetheless, advances in antiplatelet science and immunothrombosis research have created new opportunities for targeted interventions. In this section, we will review both established and emerging approaches that are of relevance to PCAS.

### 6.1. Clinically Relevant Strategies: Current Evidence and Considerations

#### 6.1.1. Antiplatelet Therapy in Post-Arrest Care

Traditional antiplatelet agents, including aspirin and P2Y_12_ inhibitors such as clopidogrel, prasugrel, and ticagrelor, are cornerstones of therapy for acute coronary syndromes [89]. Their role in PCAS, however, is not well-defined. Aspirin may mitigate platelet-driven reperfusion injury and reduce microvascular thrombosis, but evidence is limited and the bleeding risk is heightened in patients with coagulopathy, recent CPR trauma, or the need for invasive procedures.

The use of P2Y_12_ inhibitors is complicated by impaired enteral absorption, altered metabolism during shock, and delayed onset during targeted temperature management (TTM). Ticagrelor, which is not dependent on hepatic activation, may offer more reliable inhibition under hypothermic conditions, but its hemodynamic and bleeding risks require careful consideration. Overall, antiplatelet therapy must be individualized, guided by the presence of coronary occlusion, the patient’s bleeding profile, and the anticipated interventions.

#### 6.1.2. Heparin and Anticoagulation Strategies

Heparin is still widely used during post-arrest care for its anticoagulant and anti-inflammatory properties, particularly in patients requiring mechanical circulatory support [90]. Unfractionated heparin inhibits thrombin and factor Xa, preventing further microvascular thrombosis, while also attenuating leukocyte recruitment and endothelial activation. However, achieving therapeutic anticoagulation is challenging in PCAS due to fluctuating fibrinogen levels and platelet consumption and variable heparin responsiveness [91].

Low-molecular-weight heparin (LMWH) provides more predictable pharmacokinetics but is less easily titratable during invasive procedures or in the setting of a hemorrhage. Despite these limitations, anticoagulation still represents a pragmatic approach to preventing secondary thrombotic complications in carefully selected patients.

#### 6.1.3. Targeted Temperature Management (TTM) and Platelet Function

TTM, a central component of post-arrest care, exerts important modulatory effects on platelet biology. Hypothermia (32–36 °C) reduces platelet aggregation, increasing the bleeding risk while also potentially mitigating harmful thromboinflammatory activity [92]. Hypothermia also slows the hepatic metabolism, altering the pharmacokinetics of P2Y_12_ inhibitors and contributing to delayed or incomplete platelet inhibition [93].

These interactions underscore the need to integrate temperature-dependent platelet physiology into antithrombotic decision-making. Adjustments in dosing, timing, and monitoring of platelet-targeted therapies may be necessary to optimize outcomes.

#### 6.1.4. ECMO-Associated Platelet Dysfunction

ECMO, which is increasingly used in refractory cardiac arrest, profoundly alters platelet biology. Contact between blood and artificial surfaces leads to platelet activation and consumption and the shedding of receptors such as GPVI and GPIbα [94]. These changes coexist with a paradoxical hypercoagulable state driven by inflammatory activation and complement–platelet crosstalk.

Effective anticoagulation during ECMO involves balancing thrombotic risk with the prevention of bleeding, particularly in the context of platelet exhaustion or thrombocytopenia. Novel, more biocompatible ECMO circuit coatings [95] and targeted platelet-protective strategies may improve outcomes in this vulnerable population.

### 6.2. Emerging Therapeutic Targets: Modulating Thromboinflammation

#### 6.2.1. GPVI and Collagen-Driven Platelet Activation

Glycoprotein VI (GPVI) is a key receptor mediating platelet activation in response to vascular injury and collagen exposure. GPVI blockade with agents such as glenzocimab or revacept has shown promise in reducing microvascular thrombosis in ischemia–reperfusion models [96]. GPVI inhibition offers the advantage of attenuating pathological platelet activation without significantly impairing primary hemostasis, making it an appealing strategy for PCAS cases with an elevated risk of bleeding. From a translational standpoint, GPVI-targeting agents are in early clinical development, with human studies conducted in non-PCAS settings (e.g., acute ischemic stroke and other thrombotic conditions), but no dedicated PCAS trials are currently available.

#### 6.2.2. P-Selectin Blockade and Platelet–Leukocyte Crosstalk

P-selectin mediates the early adhesion between activated platelets and leukocytes. Inhibitors such as crizanlizumab, already approved for vaso-occlusive crises in sickle cell disease [97], may reduce platelet–leukocyte aggregates and downstream inflammasome activation. However, its use in PCAS remains investigational, and clinical evidence in post-cardiac arrest populations is currently lacking. By blunting the formation of immunothrombotic complexes, P-selectin inhibition may protect against microvascular obstruction and systemic inflammation in PCAS.

#### 6.2.3. Targeting Factor XI and XIa

Anticoagulants targeting factor XI or XIa—such as asundexian or milvexian—represent a new class of agents that are capable of reducing thrombosis with a substantially lower bleeding risk than traditional anticoagulants [98]. Because FXI amplifies thrombin generation without being essential in hemostasis [99,100], its inhibition may be particularly valuable in PCAS, where fragile vascular beds coexist with high thrombotic potential. Importantly, FXI/XIa inhibitors have progressed into phase II–III clinical trials for thrombotic indications (e.g., secondary stroke prevention and atrial fibrillation), but their efficacy and safety have not been established in PCAS.

#### 6.2.4. Inhibition of NETosis and Complement Pathways

Strategies aimed at suppressing neutrophil extracellular trap (NET) formation—including PAD4 inhibitors [101], DNase therapy [102], or agents targeting upstream platelet activation [103]—offer another promising avenue for mitigating microvascular obstruction. Complement inhibitors, particularly those targeting C5 [104], may also reduce platelet activation and endothelial injury, though their role in PCAS remains exploratory. At present, most NETosis-targeting approaches (e.g., PAD4 inhibition) remain preclinical or early translational, whereas complement C5 blockade is clinically approved for select hematologic/immunologic diseases but has not been systematically evaluated in PCAS.

#### 6.2.5. NLRP3 Inflammasome Modulation

Because platelet–leukocyte interactions drive NLRP3 activation and downstream IL-1β release [105], agents that modulate inflammasome activity (e.g., anakinra, colchicine, direct NLRP3 inhibitors) may attenuate systemic inflammation and end-organ injury after cardiac arrest. These interventions are especially relevant, given the central role of immunothrombosis in PCAS. Among these, anakinra and colchicine are clinically approved for inflammatory indications outside of PCAS, while direct NLRP3 inhibitors remain in early-phase clinical development, and none have been tested in dedicated PCAS trials to date.

The therapeutic modulation of platelet activation and platelet–immune crosstalk represents a promising strategy for mitigating the thromboinflammatory cascades that drive poor outcomes in PCAS. While current antiplatelet and anticoagulant therapies are limited by bleeding risk, emerging agents that selectively target pathological platelet activation—such as GPVI inhibitors, P-selectin blockers, FXI/XIa inhibitors, and NETosis modulators—provide new avenues for improving microvascular perfusion and reducing inflammation. Integrating platelet-focused strategies into post-arrest care may ultimately enhance survival and neurological outcomes, but clinical translation will require careful patient selection, physiological monitoring, and rigorous trials.

### 6.3. Feasibility and Practical Limitations in PCAS

**Figure 5:**
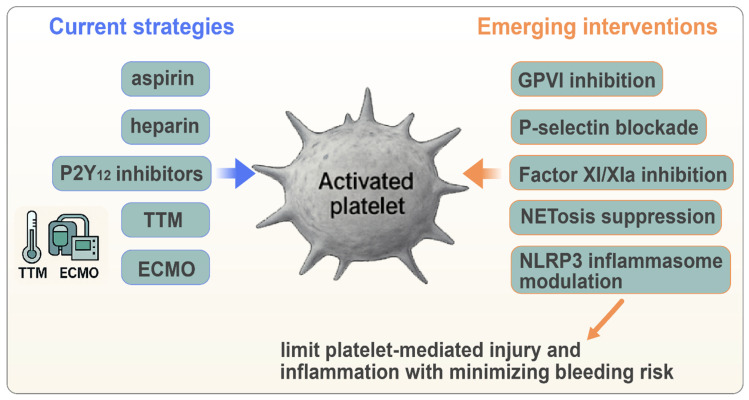
**Therapeutic strategies targeting platelet-driven thromboinflammation in post-cardiac arrest syndrome.** Overview of current and emerging interventions aimed at modulating pathological platelet activation after cardiac arrest. The central illustration shows the activated platelet as a key therapeutic target. Current strategies include aspirin, P2Y_12_ inhibitors, heparin, and supportive modalities such as targeted temperature management (TTM) and ECMO. Emerging interventions target specific thromboinflammatory pathways, including GPVI inhibition (e.g., glenzocimab), P-selectin blockade, factor XI/XIa inhibition, NETosis suppression, and NLRP3 inflammasome modulation. These targeted approaches aim to attenuate platelet-mediated microvascular injury and systemic inflammation while minimizing the bleeding risk.

In translating platelet-centered strategies to PCAS, several practical constraints must be acknowledged. First, PCAS evolves dynamically from an early reperfusion-dominant phase (minutes to hours after ROSC) to later stages that are characterized by fluctuating coagulopathy, endothelial injury, and platelet exhaustion; therefore, the therapeutic window and risk–benefit profile are likely phase-dependent. Interventions aimed at limiting microvascular obstruction and thromboinflammation may be most relevant early, yet this is also the period of highest bleeding vulnerability due to CPR-related trauma, invasive procedures, and hemodynamic instability. Second, many PCAS patients cannot reliably receive or absorb oral agents; thus, rapid-acting, titratable IV formulations (or parenteral alternatives) and bedside monitoring strategies are critical considerations. Third, the pharmacokinetics and pharmacodynamics of antithrombotic therapies are incompletely defined under TTM and ECMO. Hypothermia can impair platelet function and delay or weaken P2Y_12_ inhibition via reduced gastrointestinal motility and altered metabolism, while ECMO circuits can trigger continuous platelet activation/consumption and drug sequestration or altered clearance, complicating dosing and safety. Finally, although targets such as GPVI, P-selectin, FXI/XIa, and NETosis modulation may theoretically decouple thrombosis from hemostasis, clinical implementation in PCAS will require careful patient selection, phase-specific timing, and physiology-guided monitoring to avoid exacerbating hemorrhage or procedural complications. In contrast to anti-inflammatory therapies that primarily suppress downstream cytokine signaling, or endothelial-targeted strategies aimed at preserving vascular integrity, platelet-centered interventions uniquely target the upstream convergence of thrombosis, inflammation, and microvascular obstruction. As such, platelet-focused approaches are best viewed as complementary rather than substitutive, with the potential to synergize with the existing anti-inflammatory and endothelial-protective therapies in PCAS. Notably, these feasibility constraints are unlikely to apply uniformly across all PCAS patients, further underscoring the need for phenotype-informed therapeutic selection (Figure 5).

Collectively, while these emerging strategies offer mechanistic specificity, their translation to PCAS will require phase-specific timing, rapid-acting and titratable formulations, and careful integration with the bleeding risk, TTM, and ECMO-related platelet dysfunction, rather than a uniform post-arrest application.

## 7. Conclusions and Future Directions

Throughout this review, we distinguish associative findings from causal mechanistic evidence. Observational associations in human PCAS/ROSC cohorts (e.g., platelet biomarkers or cellular aggregates correlated with outcomes) are described as such, whereas causal inferences are drawn primarily from mechanistic animal models or well-defined experimental systems. Where direct PCAS-specific evidence is lacking, interpretations are explicitly framed as extrapolations from related ischemia–reperfusion conditions.

Platelets have long been primarily viewed as mediators of hemostasis; however, growing evidence demonstrates that they are central orchestrators of the thromboinflammatory processes that define post-cardiac arrest syndrome (PCAS). Through rapid activation during global ischemia–reperfusion, platelets propagate microvascular obstruction, amplify systemic inflammation, and contribute to endothelial dysfunction across the brain, heart, and peripheral organs. Their interactions with monocytes, neutrophils, and the complement system create a highly reactive immunothrombotic environment that worsens neurological injury, exacerbates myocardial stunning, and promotes multi-organ failure. These mechanistic insights establish platelets as pivotal drivers of PCAS pathophysiology, rather than passive markers of systemic injury.

Despite the critical role of platelets in post-arrest biology, platelet-centered strategies have not yet been fully integrated into resuscitation or post-resuscitation care. Clinically, dynamic platelet dysfunction—including early hyperactivation, receptor shedding, and, later, exhaustion—complicates management and impacts the safety and efficacy of antithrombotic therapies, targeted temperature management, and mechanical circulatory support. Platelet activation markers and platelet–leukocyte aggregates show promise as prognostic tools, yet their routine adoption remains limited. Emerging therapies that selectively modulate pathological platelet activation—such as GPVI inhibitors, P-selectin antagonists, factor XI/XIa inhibitors, and NETosis-targeting agents—offer new avenues for safeguarding microvascular perfusion while minimizing the bleeding risk. However, the translational potential of these approaches in PCAS is yet to be rigorously evaluated.

Looking ahead, integrating platelet biology into the conceptual framework of PCAS presents several opportunities. Firstly, platelet-based biomarkers may enable earlier and more precise risk stratification, guiding the intensity and timing of post-arrest interventions. Secondly, advances in platelet transcriptomics, proteomics, and metabolomics could reveal novel therapeutic targets and illuminate patient-specific patterns in thromboinflammation. Thirdly, mechanistic studies in clinically relevant models—particularly those incorporating hypothermia, ECMO, or shock physiology—are essential for understanding how therapies modulate platelet behavior under real-world conditions. Finally, randomized trials that test platelet-modulating strategies in carefully phenotyped post-arrest populations will be necessary to define their safety and efficacy and the optimal integration into the existing care pathways.

In conclusion, platelets represent a critical and underrecognized nexus linking ischemia–reperfusion injury, microvascular dysfunction, and systemic inflammation after cardiac arrest. A deeper understanding of platelet-driven mechanisms, combined with the development of targeted therapeutic approaches, holds the potential to improve survival and neurological outcomes. As this field continues to evolve, embracing platelet-centered perspectives may allow us to redefine the biological foundations and clinical management of PCAS.

## Figures and Tables

**Table 1:** Pathways with direct human PCAS evidence vs. extrapolated evidence.

Pathway/Mechanistic Theme	Representative Readouts/Endpoints	Direct Human PCAS Evidence	Evidence Level
Consumptive thrombocytopenia/platelet consumption after ROSC	Early thrombocytopenia due to adhesion, sequestration, microthrombi	Yes	Human PCAS clinical observation
Global coagulation phenotype (TEG/ROTEM)	Hyper- or hypocoagulable patterns post-ROSC	Yes	Human critical-care phenotype
Platelet morphometrics (MPV/PDW)	Elevated MPV/PDW correlating with outcomes	Yes	Human prognostic association
Soluble platelet activation markers	sP-selectin, sCD40L elevation after ROSC	Yes	Human PCAS biomarker data
Platelet–leukocyte aggregates	Increased PLAs correlating with lactate, organ failure, mortality	Yes	Human PCAS activation phenotype
NETosis and platelet–neutrophil crosstalk	NET-mediated microvascular obstruction	Limited	Mainly extrapolated
Platelet-derived microparticles	PMP surge after cardiac arrest	Yes	Human post-arrest increase
TTM–platelet interaction	Hypothermia alters aggregation and P2Y_12_ PK/PD	Yes	Human post-arrest therapy context
ECMO-associated platelet dysfunction	Activation, consumption, receptor shedding	Contextual	Largely extrapolated from the ECMO literature

## Data Availability

The original contributions presented in this study are included in the article. Further inquiries can be directed to the corresponding authors.
